# Service-Delivery Models to Increase the Uptake of Non-Communicable Disease Screening in South-Central Ethiopia: A Difference-In-Differences Analysis

**DOI:** 10.3390/diseases12110278

**Published:** 2024-11-05

**Authors:** Bezawit Ketema, Adamu Addissie, Sarah Negash, Mosisa Bekele, Andreas Wienke, Mirgissa Kaba, Eva Johanna Kantelhardt

**Affiliations:** 1 School of Public Health, College of Health Sciences, Addis Ababa University, 9086 Addis Ababa, Ethiopia; 2 Institute for Medical Epidemiology, Biostatistics and Informatics, Martin Luther University, 06112 Halle, Germany; 3 Global Health Working Group, Martin Luther University, 06097 Halle, Germany

**Keywords:** non-communicable disease, difference-in-differences, social- and behavioral-change communication, screening, effectiveness

## Abstract

Background: Screening for non-communicable diseases (NCDs) is a critical step for early detection and the prevention of consequent morbidity and mortality. To facilitate NCD screening, the Ethiopian Ministry of Health has developed screening guidelines. However, like other low- and middle-income countries, interventions to increase the uptake of NCD-screening services in Ethiopia remain ineffective. Thus, this study aimed to determine the effectiveness of service delivery models to increase NCD-screening service uptake in south-central Ethiopia. Method: A health-facility-based quasi-experimental study design was employed to determine the effectiveness of providing a multiple-NCD-screening service in addition to social- and behavioral-change communication (SBCC) intervention to increase the uptake of NCD-screening services. The interviewer-administered structured questionnaire was adapted from previously published research and used to collect data during the baseline and end-line survey periods. A difference-in-differences analysis was used to determine the effectiveness of the intervention. Results: Compared with routine care, the availability of a multiple-NCD-screening service, together with SBCC intervention, was found to significantly increase the uptake of cervical cancer screening, clinical breast examination, blood pressure measurement, and blood glucose-measurement services, by 18, 9, 44 and 23 percent points, respectively. However, the availability of a multiple-NCD-screening service without SBCC intervention increased clinical breast-examination service uptake by 9% point and blood glucose-measurement service uptake by 18% point without increasing the uptake of cervical cancer-screening or blood pressure-measurement services. Conclusion: The integration of multiple-NCD-screening services accompanied by SBCC intervention that promotes them is an important approach for improving the uptake of NCD-screening services.

## 1. Introduction

Non-communicable diseases (NCDs) kill 41 million people each year, which is equivalent to 74% of all deaths globally [1]. Every 2 s, someone aged 30 to 69 dies prematurely from an NCD [2]. The main types of NCDs are cardiovascular diseases, cancers, chronic respiratory diseases, and diabetes. These diseases disproportionately affect people in low- and middle-income countries, where more than three quarters of global NCD deaths occur [3]. In Ethiopia, NCDs accounted for 42% of the total deaths in the country in 2022 [4].

The aim of Sustainable Development Goal Target 3.4 is to reduce premature mortality from NCDs by one third by 2030 [5]. In 2017, the 70th World Health Assembly endorsed an updated list of interventions for NCD prevention and control, which included screening as a key strategy [6]. Likewise, the Ethiopian national guidelines on NCDs aim to limit the rising burden of NCDs through a three-tiered prevention strategy: primary prevention via vaccinations; secondary prevention through screening; and tertiary prevention through diagnosis, treatment, and care [7].

However, evidence indicates that NCD-screening service uptake is lower in low- and middle-income countries [8], including in Ethiopia [9,10,11,12], compared with high-income nations [13]. A national survey conducted in Ethiopia in 2015 revealed that only 3%, 23.4%, and 2.65% of the eligible population had ever undergone blood glucose measurement, blood pressure measurement, and cervical cancer screening, respectively [14].

Various factors contributed to the low NCD-screening service uptake in Ethiopia. One qualitative study found that a lack of awareness, poor health-seeking behaviors, profound social consequences and exclusion, and limited access to services were reasons behind the poor uptake of cervical cancer screening [15]. Similar reasons have been identified as barriers to the early diagnosis of breast cancer [16].

Moreover, there is evidence that NCDs commonly show comorbidity. A population-based prospective cohort study found that genetically instrumented type 2 diabetes mellitus was associated with an increased risk of hypertension (AOR = 1.07; 95% CI: 1.04–1.10) [17]. This study recommends regular blood pressure measurement, together with blood glucose measurement. A systematic review and meta-analysis revealed a statistically significant association between hypertension and increased risk of breast cancer (RR = 1.15; 95% CI: 1.08–1.22) [18]. Another systematic review indicated that diabetes was related to poorer overall survival (HR = 1.59, 95% CI: 1.35–1.87) and poorer recurrence-free survival (HR = 1.98, 95% CI: 1.47–2.66) in cervical cancer patients [19]. Given the interrelated nature of these NCDs, a multiple-screening approach, in which individuals are screened simultaneously for multiple NCDs, is recommended in a variety of settings [20,21].

However, studies on multiple-NCD screening in Ethiopia are limited. Thus, this study aimed to determine the effectiveness of service delivery models of multiple-NCD screening to increase the uptake of NCD-screening services in south-central Ethiopia using a difference-in-differences (DiD) analysis. Findings from this study could inform policy makers and enable program planners to develop optimal strategies to increase the uptake of NCD-screening services in Ethiopia and similar settings.

## 2. Materials and Methods

### 2.1. Study Design and Study Area

A health-facility-based quasi-experimental study design was employed to determine the effectiveness of service delivery models to increase NCD-screening service uptake. This study was conducted at primary healthcare facilities (PHFs) in the southwest Shewa zone of the Oromia region, and in the Hadiya and Gurage zones of the Central Ethiopia region. Ethiopia is a federation that is subdivided in to ethno-linguistically based regional states and chartered cities. There are 12 regional states and 2 chartered cities in Ethiopia. According to the Ethiopian Central Statistical Agency Population Projection of 2024, Oromia and Central Ethiopia are the first and third most populous regions in the country [22]. Each region in Ethiopia is subdivided into zones, which are the next smallest administrative structures.

### 2.2. Study Process and Study Period

This study was conducted in two phases: baseline and endline phases. The current uptake of NCD-screening services (cervical cancer screening, clinical breast examination, blood pressure measurement, and blood glucose measurement) was measured during the baseline- and endline-survey times. The baseline survey was administered before the commencement of this study intervention, between 10 October and 10 November 2020. The endline survey took place from 20 December 2021, to 20 January 2022, after the intervention was introduced within the intervention facilities.

There were three arms in this study; the component-intervention arm, the single-intervention arm, and the control arm. Each healthcare facility included in this study was allocated to one of the three arms. Healthcare facilities allocated to the component-intervention arm engaged with two intervention components: a multiple-NCD-screening service, in addition to social–behavioral change communication (SBCC) promoting the NCD screening. Healthcare facilities allocated to the single-intervention arm engaged in a single intervention: the multiple-NCD-screening service. Arm three was the control arm of the study, consisting of the routine NCD-screening service; neither the multiple-NCD screening nor the SBCC intervention of this study was delivered to the healthcare facilities in the control arm.

### 2.3. Descriptions of Routine Care and Intervention Care

Routine care: based on the National NCD Prevention and Control Guideline of Ethiopia, PHFs are expected to provide cervical cancer screening, clinical breast examination, blood pressure measurement, and blood glucose measurement within the available screening services. In addition, PHFs are required to provide awareness-creation programs on NCDs via SBCC, mainly through mass communication [23]. As standard care at PHFs, cervical cancer screening and clinical breast examination are being offered together, in a room labeled “Cervical Cancer Screening Unit” [24]. A blood pressure (BP)-measurement service is being offered at the out-patient department, and blood glucose measurement is performed in the laboratory [7]. Cervical cancer screening, clinical breast examination, and BP measurement are provided free of charge, but patients are required to pay USD 0.5 for the blood glucose-measurement service that is part of the routine care at PHFs [7,24].

Intervention care: for this study, a multiple-NCD-screening service is defined as providing an NCD-screening corner at the healthcare facilities at which all NCD screening assessments—for cervical cancer, clinical breast examination, BP, and blood glucose—are offered. Thus, based on their eligibility, participants could take up multiple NCD-screening services at once. All four screening services in the intervention facilities were free of charge. For this study, SBCC is defined as promoting NCD screening via different channels within the healthcare facility. The channels considered for this purpose were audiovisual and print media, which included posters, leaflets, and a billboard. The audiovisual component shows healthcare professionals encouraging regular NCD screening, along with testimonials from survivors emphasizing the benefits of early detection. Posters stress the ease of NCD screening, the often asymptomatic nature of NCDs, and the importance of a healthy lifestyle. Leaflets give definitions of NCDs, their risk factors, potential complications, and preventative measures. The billboard visually shows the NCD-screening procedures being targeted.

### 2.4. Population

Eligible adults who visited selected healthcare facilities in the study period formed the study population. All sampled adults who attended the selected healthcare facilities during the study period and fulfilled the inclusion criteria were included as participants in the study. The inclusion criteria were as follows: female aged 30–49 years for cervical cancer screening, female aged ≥ 18 years for clinical breast examination, male or female aged ≥ 30 years for BP measurement, and male or female ≥ 45 years for blood glucose measurement. The criteria was set according to the Ethiopian national NCD-screening guidelines [7]. However, individuals with any type of confirmed NCD, admitted patients, and emergency case patients were all excluded from this study.

### 2.5. Sample-Size Determination

The required sample size was determined independently for the four outcome variables (cervical cancer screening, clinical breast examination, BP measurement, and blood glucose measurement) using the double-population proportion formula in OpenEpi version 7 software. The following assumptions were made: a proportion of 2.65% was considered independently for cervical cancer-screening and clinical breast-examination service uptakes. A proportion of 3% was considered independently for BP measurement and blood glucose-measurement service uptakes [14]. Proportions of 2.65% and 3% were assumed for the control arm. A difference in proportions with the component-intervention arm and the control arm was assumed to be 20%, and for the single-intervention and control arm it was assumed to be 10%; it was supposed that the intervention would increase the screening uptake by 20% point in the component-intervention arm and by 10% point in the single-intervention arm. The ratio of unexposed to exposed group was considered to be 3. A level of confidence of 95%, a 15% lost to follow-up, and a design effect of 2 were considered.

The calculated sample size for cervical cancer screening was the following: for component-intervention arm to control-arm analysis it was 69, 206 (N = 274), and for single-intervention arm to control-arm analysis it was 174, 523 (N = 697). The calculated sample size for BP measurement was the following: for component-intervention arm to control-arm analysis it was 70, 210 (N = 280), and for single-intervention arm to control-arm analysis it was 181, 543 (N = 724). Sample size for clinical breast examination was considered similar to that for cervical cancer screening, and sample size for blood glucose measurement was considered similar to that for blood pressure measurement.

### 2.6. Sampling Technique

Locations of the three arms of this study were selected by considering market areas, physical access, population movement, and buffer areas to protect the sharing of information between the study arms. PHFs that ensured the availability of routine cervical cancer-screening, clinical breast examination, BP-measurement, and blood glucose-measurement services during the data collection period were considered for inclusion in this study. Four PHFs were selected per study arm, yielding a total of 12 PHFs in this study. After the PHFs had been selected, individual study participants from each PHF were selected, using a systematic random-sampling technique.

### 2.7. Measurements and Data Collection

The study data were collected using an online data collection kit (ODK), at the exit gate of selected healthcare facilities after participants completed their stay at the facility. A structured interviewer-administered questionnaire was adapted from previous studies [25,26,27]. The questionnaire was developed in the English language, translated into local languages (Amharic and Afan Oromo), and then translated back into English to maintain consistency in meaning and sense, as determined by language experts. The first draft of the questionnaire was pilot-tested and appropriate modifications were made to improve clarity, question ordering, and the nature of the questions.

As an outcome variable for this study, participants were asked yes/no questions about whether they had taken up NCD-screening services during their current facility visit. The following explanatory variables were included: socio-demographic variables (age, sex, marital status, educational status, and occupation); knowledge about cervical cancer, breast cancer, hypertension, and diabetes mellitus; and previous uptake of cervical cancer screening, clinical breast-examination, BP-measurement, and blood glucose-measurement services.

Knowledge about cervical cancer was assessed by 12 items (5 items about risk factors, 4 items about signs and symptoms, and 3 items about prevention). Knowledge about breast cancer was assessed by 26 items (7 items about risk factors, 9 items about signs and symptoms, and 10 items about prevention). Knowledge about hypertension was assessed by 16 items (6 items about risk factors, 5 items about signs and symptoms, and 5 items about prevention). Knowledge about diabetes mellitus was assessed by 17 items (5 items about risk factors, 6 items about sign and symptoms, and 6 items about prevention) (Table S1). Scores from knowledge-measuring items addressing a specific NCD were summed and treated as a continuous variable. Then, the mean scores were computed to determine the overall knowledge status of the participants for each NCD. Average and above-average scores were defined as knowledgeable about the relevant NCD, whereas below-average scores were defined as not knowledgeable. Each participant’s previous uptake of screening services for the different NCDs was assessed separately, using questions requiring yes/no answers.

### 2.8. Data Processing and Analysis

The responses in the completed ODK were exported to STATA version 17 for analysis. Descriptive statistics were calculated for the intervention and control groups at baseline. A split DiD analysis was used to determine the effect of the intervention separately on cervical cancer-screening, clinical breast-examination, BP-measurement, and blood glucose-measurement service uptakes. We employed the DiD analysis using variables describing socio-demographic characteristics, knowledge, and previous screening service uptake as covariates using a t-statistical test. The DiD estimates in this study measured the changes in the outcome variables (uptake of cervical cancer screening, clinical breast examination, BP measurement, and blood glucose measurement), which resulted from the intervention. Unadjusted and adjusted DiD estimates were calculated to account for differences in participant characteristics between groups.

## 3. Results

### 3.1. Socio-Demographic Characteristics of Study Participants

In this study, at baseline, 681, 559, and 835 participants participated in the component-intervention arm, single-intervention arm, and control arm, respectively, which gave an adequate power of greater than 80%.

The mean (±SD) age in years of study participants was 43.4 (±11.1), 45.1 (±8.8), and 44.8 (±10), respectively, in the component-intervention arm, single-intervention arm, and control arm. Across all of the study arms, the majority of participants were females, most of whom were married. The majority of study participants in the component-intervention arm, single-intervention arm, and control arm could not read and write. Regarding occupation, the majority of participants were housewives in the component-intervention arm (364; 53%) and the control arm (516; 62%), whereas the majority of participants in the single-intervention arm (384; 69%) were farmers (Table 1).

### 3.2. NCD-Screening Service Uptake Measured in Baseline and Endline Surveys

**Figure 1:**
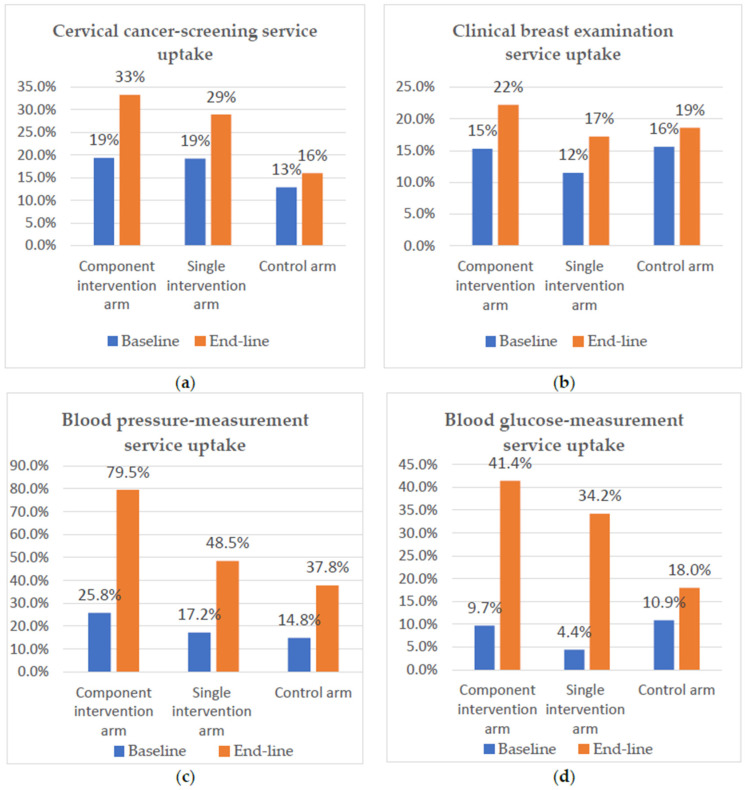
(**a**) Proportion of cervical cancer-screening service uptake; (**b**) proportion of clinical breast-examination service uptake; (**c**) proportion of BP-measurement service uptake; and (**d**) proportion of blood glucose-measurement service uptake. The component-intervention arm included a multiple-NCD-screening service plus social- and behavioral-change communication intervention. The single-intervention arm included the multiple-NCD-screening service only.

The proportion of cervical cancer-screening service uptake among the study participants in the component-intervention arm increased from 19% at baseline to 33% at endline. Similarly, in the single-intervention arm, cervical cancer-screening service uptake increased from 19% to 29%, whereas in the control arm it was 13% at baseline and 16% at endline. The proportion of clinical breast-examination service uptake increased from 15% at baseline to 22% at endline in the component-intervention arm; similarly, in the single-intervention arm it rose from 12% to 17%. The proportion of clinical breast-examination service uptake in the control arm also increased, going from 16% at baseline to 19% at endline (Figure 1).

The proportion of BP-measurement service uptake in the component-intervention arm increased from 26% at baseline to 79% at endline, and increased in the single-intervention arm from 17% to 48%. On the other hand, the proportion of BP-measurement service uptake increased from 15% to 38% in the control arm. Blood glucose-measurement service uptake increased from 10% at baseline to 41% at endline in the component-intervention arm, increased from 4% to 34% in the single-intervention arm, and increased from 11% to 18% in the control arm (Figure 1).

### 3.3. NCD-Screening Service Uptake According to Socio-Demographic Characteristics, Knowledge, and Previous Screening Service Uptake Within Study Arms at Endline Survey

Across all study arms, over three-quarters of women who had taken up cervical cancer screening at the time of the endline survey were married, reflecting a similar trend for clinical breast-examination service uptake. In the control arm, most women who underwent cervical cancer screening (CCS) had completed at least secondary education (43%) and were employed (48%). Conversely, women in the component- and single-intervention arms were more likely to be illiterate (48% and 65%, respectively) and engaged in domestic work (53% and 49%). Similar trends have been observed among those who underwent clinical breast examination at the endline survey; women in the control arm were predominantly educated and employed, while those in the intervention arms were more likely to be illiterate, and housewives or farmers. Notably, women in both intervention arms were mostly knowledgeable about cervical cancer and breast health. Furthermore, a significant proportion of women across all arms had no prior history of clinical breast examination (65%, 78%, and 99% in the component-intervention, single-intervention, and control arms, respectively). However, in the control arm, the majority of women (85%) had previously undergone CCS (Table 2).

Across all study arms, the majority of participants who received BP measurement services were female. However, a different trend emerged for blood glucose measurement, with males representing the majority of participants in all arms (52%, 59%, and 53%). The majority of participants who underwent BP measurement and/or blood glucose measurement were married or living together. In the control arm, those who received BP measurements were predominantly educated at the secondary level or higher (37%). In contrast, participants in the intervention arms were more likely to be illiterate, with frequencies of 60% for both component-intervention and single-intervention arms. Overall, a significant proportion of participants in all arms demonstrated knowledge about hypertension and diabetes diseases, but very few had prior experience of BP-measurement and blood glucose-measurement services (Table 3).

### 3.4. NCD-Screening Service Uptake Among Participants Eligible for All Available Screening Services at the Endline-Survey Visit

**Figure 2:**
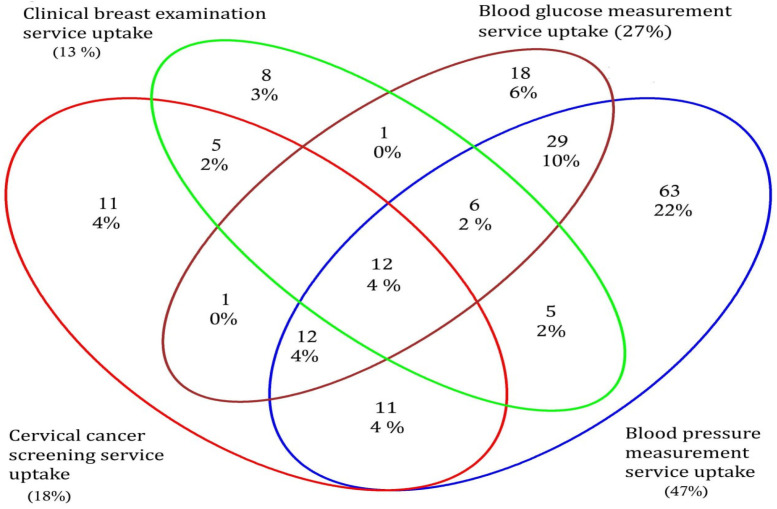
NCD-screening service uptake among participants eligible for all target NCDs during the endline-survey visit (n = 293).

As per the inclusion criteria, a total of 293 endline-survey participants were eligible to take up all of the four screening services at the endline-survey visit. From those, 52 (18%) took up CCS, 37 (13%) took up clinical breast examination, close to half 138 (47%) of participants took up BP measurement, and 79 (27%) took up blood glucose-measurement service at the endline-survey visit. Very few study participants, 12 (4%), took all of the available NCD-screening services. Even though they were eligible for all of the available NCD-screening services, more than one third, 111 (38%), of the study participants did not take up any of the screening services (Figure 2).

### 3.5. Effect of Multiple NCD-Screening Service Availability with SBCC on NCD-Screening Service Uptake

Compared with routine care, the availability of a multiple-NCD-screening service accompanied by SBCC (component intervention) increased NCD-screening service uptake. The intervention significantly increased the uptake of CCS, clinical breast examination, BP measurement, and blood glucose measurement by 18, 9, 44, and 23 percent points, respectively (Table 4).

### 3.6. Effect of Multiple NCD-Screening Service Availability Without SBCC on NCD-Screening Service Uptake

Compared with the routine care, the availability of a multiple NCD-screening service without accompanying SBCC (single intervention) had effect on increasing clinical breast-examination service uptake and blood glucose-measurement service uptake. The intervention increased clinical breast-examination service uptake by 9% point and blood glucose-measurement service uptake by 18% point. Nevertheless, CCS service uptake and BP-measurement service uptake were not affected (Table 5).

## 4. Discussion

In our study, compared with routine care, providing a multiple-NCD-screening service together with SBCC interventions (the component intervention) increased the uptake of all targeted NCD-screening services (CCS, clinical breast examination, BP measurement, and blood glucose measurement). This implies that a component-intervention approach is effective in increasing NCD-screening service uptake. In line with this, a systematic review of twenty studies showed that multicomponent interventions were effective in increasing screening service uptake [28].

Compared with routine care, the availability of a multiple-NCD-screening service without SBCC intervention (the single intervention) did not show a significant effect on CCS service uptake or BP-measurement service uptake. However, a systematic review and meta-analysis to identify effective interventions to increase the uptake of CCS showed that single interventions may increase the uptake of CCS, compared with a control group [29]. This variation could be due to differences in target interventions; in our single intervention, the intervention was providing an NCD-screening corner at the healthcare facilities at which combined screening and measurement services for all NCDs—cervical cancer, clinical breast examination, BP, and blood glucose—were being offered. However, the interventions in the studies that were included in the systematic review and meta-analysis described above were counseling, health education, reminders, invitations, messaging, economic intervention (e.g., subsidized cost), and procedure provision (e.g., HPV testing, visual inspection with acetic acid (VIA) test, etc.). Moreover, according to a scoping review of the literature and resources for CCS interventions, no associations with effect size were noted for a number of intervention components [30]. Different health-behavior-change theories and models such as the health belief model [31] and the theory of planned behavior [32] indicated that behavior-change communication interventions would facilitate the occurrence of healthy behaviors. Research also indicated that effective health-communication interventions can promote positive behavior change and help to prevent diseases [33,34].

The current study showed that the uptake of all NCD-screening services increased in all arms over time from base-line to endline-survey times. The service uptake increment in the control arm could be due to the fact that people are constantly learning, both intentionally and unintentionally, throughout their lives, which naturally increases the uptake over time. In the control arm of this study, BP-measurement service uptake showed a greater increase (23%) compared with the other target NCD-screening services. This could be due to the fact that there was an active effort to improve hypertension prevention and control at the primary health-care level in Ethiopia during this study period. This effort was made by the Federal Ministry of Health of Ethiopia in collaboration with the World Health Organization (WHO) and Resolve to Save Lives [35].

Our findings in the control arm at the endline-survey visit suggested that the majority of women who underwent clinical breast examination and/or CCS were at a secondary educational level or above, and were mostly employed. Similar to this finding, other studies have also reported positive associations between educational status and CCS service uptake [36,37,38]. Another study conducted in Ethiopia also showed that employed women were likely to undergo breast screening [39]. However, in both the component-intervention and single-intervention arms of this study, most women who underwent clinical breast examination at the endline survey could not read and write, and were housewives. This implies the interventions in this study were important in reaching less-educated and non-employed women for better uptake of clinical breast examination and/or CCS services.

In this study, most women who underwent clinical breast examination or/and CCS across all of the arms were those who were knowledgeable about the disease. This positive association between knowledge and screening service uptake had been reported in many other studies, as well [40,41,42,43]. This indicates that educational interventions may be helpful in increasing NCD-screening service uptake.

The majority of the participants who took up screening services (CCS, clinical breast examination, and blood glucose measurement) in the intervention arms of this study were those who had not taken it up previously. However, in most other scenarios, people would likely take up the screening service when they had experienced it before [44,45]. The same was true in the control arm of this study, in which the majority of the women who underwent CCS were those who have taken it up previously. Thus, this study intervention could be recommended to motivate people to take up the screening service, even if they had not had this experience before.

In this study, a total of 293 participants were eligible to take up all of the targeted NCD-screening services (CCS, clinical breast examination, BP measurement, and blood glucose measurement) at the endline-survey visit. Among the 293 endline-survey participants who were eligible for all screening services, 133 (47%) participants had taken up the BP-measurement service by the endline-survey visit. This could be due to the fact that BP measurement is the simplest procedure of the screening procedures used in this study. Among those participants who were eligible for all screening services, very few of them, 12 (4%), had taken up all of the available NCD-screening services by the time they completed the endline-survey visit. This could mean that, even if NCD-screening services are available in an integrated way, their uptake would vary, based on the available test strategies and procedures of each target NCD.

In general, in this study, we found that delivering different NCD-screening services (CCS, clinical breast examination, BP measurement, and blood glucose measurement) in an integrated way, together with SBCC efforts at PHFs, significantly increased the uptake of screening services. Our finding was in line with the WHO’s package of essential non-communicable-disease intervention recommendations for PHFs, which recommends the use of an integrated approach to NCD screening for low-resource settings [46].

### Strengths and Limitations of the Study

This study has definite strengths and limitations. As this study is a facility-based study, its findings might not be representative of the general population. In this study, more females participated than males, due to the eligibility criteria to undergo the screening services, which may limit the male representativeness of the findings.

## 5. Conclusions

Integration of the currently scattered NCD-screening services, by providing a multiple-NCD-screening service together with SBCC interventions, improves the uptake of NCD-screening services among PHF attendants. Thus, we recommend that PHFs allocate a specific department to offer a screening service targeting the main NCDs by performing the available test procedures together, and that they promote its uptake through various SBCC efforts.

## Figures and Tables

**Table 1:** Socio-demographic characteristics of study participants at baseline.

Variable	Response Categories	Component-Intervention Arm	Single-Intervention Arm	Control Arm
		(n = 681)	(n = 559)	(n = 835)
Age in years	(mean ± SD)	43.4 (±11.1)	45.1 (±8.8)	44.8 (±10)
Sex	Female	558 (82%)	417 (75%)	727 (87%)
	Male	123 (18%)	142 (25%)	109 (13%)
Marital status	Married	555 (82%)	507 (91%)	534 (64%)
	Non-married	126 (19%)	52 (9%)	301 (36%)
Educational status	Secondary and above	131 (19%)	82 (15%)	85 (10%)
	Primary	125 (18%)	72 (13%)	56 (7%)
	Only read and write	129 (19%)	86 (15%)	136 (16%)
	Cannot read and write	196 (44%)	319 (57%)	558 (67%)
Occupation	Employed	157 (23%)	89(16%)	138 (17%)
	Housewife	364 (53%)	80 (14%)	516 (62%)
	Farmer	133 (20%)	384 (69%)	146 (17%)
	Unemployed	28 (4%)	6 (1%)	35 (4%)

**Table 2:** Participants who took up CCS and clinical breast-examination services during the endline-survey visit.

Variables	Response Categories	Have Taken Up CCS Service			Have Taken Up Clinical Breast Examination Service		
		Comp.-int. Arm (n = 120)	Single-int. Arm (n = 104)	Control Arm (n = 84)	Comp.-int. Arm (n = 80)	Single-int. Arm (n = 73)	Control Arm (n = 98)
Marital status	Married Unmarried	94 (78%) 26 (22%)	99 (95%) 5 (5%)	76 (91%) 8 (10%)	63 (79%) 17 (21%)	70 (96%) 3 (4%)	83 (85%) 15 (15%)
Educational status	Secondary and above Primary Only read and write Cannot read and write	14 (12%) 11 (9%) 38 (32%) 57 (48%)	9 (9%) 23 (22%) 4 (4%) 68 (65%)	36 (43%) 23 (27%) 20 (24%) 5 (6%)	11 (14%) 13 (16%) 21 (26%) 35 (44%)	6 (8%) 12 (16%) 6 (8%) 49 (67%)	55 (56%) 31 (32%) 0 (0%) 12 (12%)
Occupational status	Employed Housewife Farmer Unemployed	25 (21%) 64 (53%) 30 (25%) 1 (1%)	10 (10%) 43 (41%) 51 (49%) 0 (0%)	40 (48%) 32 (38%) 12 (14%) 0 (0%)	17 (21%) 46 (58%) 16 (20%) 1 (1%)	9 (12%) 34 (47%) 29 (40%) 1 (1%)	44 (45%) 26 (27%) 20 (20%) 8 (8%)
Knowledge	Not knowledgeable Knowledgeable	35 (29%) 85 (71%)	14 (14%) 88 (85%)	5 (6%) 79 (91%)	28 (35%) 52 (65%)	28 (38%) 45 (62%)	12 (12%) 86 (88%)
Previous screening-service uptake	Have ever had Never had	59 (49%) 61 (51%)	51 (49%) 53 (51%)	13 (16%) 71 (85%)	52 (65%) 28 (35%)	57 (78%) 16 (22%)	97 (99%) 1 (1%)

Comp.-int. arm (multiple-NCD-screening service plus social- and behavioral-change communication), single-int. arm (multiple-NCD-screening service only).

**Table 3:** Participants who took up blood pressure-measurement and blood glucose-measurement services during the endline-survey visit.

Variables	Response Categories	Have Taken Up Blood Pressure-Measurement Service			Have Taken Up Blood Glucose-Measurement Service		
		Comp.-int. Arm (n = 449)	Single-int. Arm (n = 305)	Control Arm (n = 237)	Comp.-int. Arm (n = 149)	Single-int. Arm (n = 123)	Control Arm (n = 98)
Sex	Male Female	124 (28%) 325 (72%)	97 (32%) 208 (68%)	60 (25%) 177 (75%)	78 (52%) 71 (48%)	73 (59%) 50 (41%)	52 (53%) 46 (47%)
Marital status	Married Unmarried	352 (78%) 97 (22%)	282 (93%) 23 (8%)	197 (83%) 40 (17%)	106 (71%) 43 (29%)	109 (89%) 14 (11%)	75 (77%) 23 (24%)
Educational status	Secondary and above Primary Only read and write Cannot read and write	47 (11%) 56 (13%) 76 (17%) 270 (60%)	39 (13%) 47 (15%) 35 (12%) 184 (60%)	88 (37%) 25 (11%) 52 (22%) 72 (30%)	7 (5%) 20 (13%) 21 (14%) 101 (68%)	18 (15%) 10 (8%) 12 (10%) 83 (68%)	15 (15%) 12 (12%) 12 (12%) 57 (58%)
Occupational status	Employed Housewife Farmer Unemployed	72 (16%) 209 (47%) 166 (37%) 2 (0.5%)	36 (12%) 82 (27%) 185 (61%) 1 (0.3%)	111 (47%) 76 (32%) 46 (19%) 4 (2%)	13 (9%) 35 (24%) 101 (68%) 0 (0%)	17 (14%) 16 (13%) 89 (72%) 1 (1%)	41 (42%) 16 (16%) 39 (39%) 2 (2%)
Knowledge	Not knowledgeable knowledgeable	60 (13%) 389 (87%)	69 (23%) 236 (77%)	78 (33%) 159 (67%)	49 (33%) 100 (68%)	62 (50%) 61 (50%)	54 (55%) 44 (45%)
Previous screening-service uptake	Ever had Never had	2 (0.4%) 447 (100%)	25 (8%) 280 (92%)	0 (0%) 237 (100%)	37 (25%) 112 (75%)	65 (53%) 58 (47%)	0 (0%) 98 (100%)

Comp.-int. arm (multiple-NCD-screening service plus social- and behavioral-change communication intervention), single-int. arm (multiple-NCD-screening service only).

**Table 4:** Effect of multiple-NCD-screening service availability with SBCC on NCD-screening service uptake.

Type of Service Uptake	DiD Estimates			
	Crude DiD (95% CI)	*p*-Value	Adjusted DiD * (95% CI)	*p*-Value
Cervical cancer screening	0.176 (0.130–0.221)	<0.001	0.182 (0.126–0.238)	0.015
Clinical breast examination	0.056 (0.012–0.100)	0.012	0.092 (0.009–0.176)	0.045
Blood pressure measurement	0.527 (0.485–0.569)	<0.001	0.437 (0.049–0.824)	0.044
Blood glucose measurement	0.281 (0.238–0.324)	<0.001	0.227 (0.094–0.361)	0.029

* Adjusted for age, sex, marital status, educational status, occupation, knowledge, and previous screening service uptake.

**Table 5:** Effect of opportunistic multiple-NCD-screening service availability without SBCC on NCD-screening service uptake.

Type of Service Uptake	DiD Estimates			
	Crude DiD (95% CI)	*p*-Value	Adjusted DiD * (95% CI)	*p*-Value
Cervical cancer screening	0.132 (0.088–0.177)	<0.001	0.132 (−0.084–0.347)	0.082
Clinical breast examination	0.016 (−0.024–0.055)	0.436	0.094 (0.089–0.099)	0.003
Blood pressure measurement	0.240 (0.199–0.282)	<0.001	0.230 (−0.189–0.649)	0.091
Blood glucose measurement	0.222 (0.180–0.263)	<0.001	0.182 (0.046–0.318)	0.037

* Adjusted for age, sex, marital status, educational status, occupation, knowledge, and previous screening service uptake.

## Data Availability

The data presented in this study are available on request from the corresponding author.

## Supplementary Materials

The following supporting information can be downloaded at: https://www.mdpi.com/article/10.3390/diseases12110278/s1, Table S1: A questionnaire to assess Non-Communicable Disease screening service uptake.

## References

[1] WHO (2018). Noncommunicable Diseases Country Profiles 2018.

[2] WHO (2018). World Health Organization Non-Communicable Disease Fact Sheet 2018.

[3] UNDoP (2011). Non-Communicable Diseases Deemed Development Challenge of ‘Epidemic Proportions’.

[4] Health NDMCf (2023). Burden of Non-Communicable Diseases (NCD) in Ethiopia. https://ndmc.ephi.gov.et/download/burden-of-non-communicable-diseases-ncd-in-ethiopia-2/.

[5] UN (2015). Sustainable Development Goals.

[6] WHO Preparation for the Third High-level Meeting of the General Assembly on the Prevention and Control of Non-Communicable Diseases, to be Held in 2018. Proceedings of the 17th World Health Assembly.

[7] WHO (2016). Guidelines on Clinical and Programmatic Management of Major Non Communicable Diseases.

[8] Martinez M.E., Schmeler K.M., Lajous M., Newman L.A. (2024). Cancer Screening in Low- and Middle-Income Countries. Prev. Risk Reduct. Genet..

[9] Bayu H., Berhe Y., Mulat A., Alemu A. (2016). Cervical Cancer Screening Service Uptake and Associated Factors among Age Eligible Women in Mekelle Zone, Northern Ethiopia, 2015: A Community Based Study Using Health Belief Model. PLoS ONE.

[10] Gebru Z., Gerbaba M., Dirar A. (2016). Utilization of Cervical Carcinoma Screening Service and Associated Factors among Currently Married Women in Arba Minch Town, Southern Ethiopia. J. Women’s Health Care.

[11] Ketema B., Addissie A., Negash S., Kantelhardt E.J., Kaba M. (2024). Does Prior Experience Matter? Intention to Undergo Cervical Cancer Screening among Rural Women in South-Central Ethiopia. Curr. Onclol..

[12] Ketema B., Kaba M., Negash S., Addissie A., Kantelhardt E.J. (2023). Intention to Undergo Clinical Breast Examination and Its Associated Factors among Women Attending Rural Primary Healthcare Facilities in South Central Ethiopia. Breast Care.

[13] Bruni L., Serrano B., Roura E., Alemany L., Cowan M., Herrero R., Poljak M., Murillo R., Broutet N., Riley L.M. (2022). Cervical cancer screening programmes and age-specific coverage estimates for 202 countries and territories worldwide: A review and synthetic analysis. Lancet Glob. Health.

[14] Ethiopia Public Health Institute (2016). Ethiopia Steps Report on Risk Factors for Non-Communicable Diseaes and Prevalence of Selected NCDs.

[15] Birhanu Z., Abdissa A., Belachew T., Deribew A., Segni H., Tsu V., Mulholland K., Russell F.M. (2012). Health seeking behavior for cervical cancer in Ethiopia: A qualitative study. Int. J. Equity Health.

[16] Getachew S., Tesfaw A., Kaba M., Wienke A., Taylor L., Kantelhardt E.J., Addissie A. (2020). Perceived barriers to early diagnosis of breast Cancer in south and southwestern Ethiopia: A qualitative study. BMC Women Health.

[17] Sun D., Zhou T., Heianza Y., Li X., Fan M., Fonseca V.A., Qi L. (2019). Type 2 Diabetes and Hypertension A Study on Bidirectional Causality. AHA J..

[18] Han H., Guo W., Shi W., Yu Y., Zhang Y., Ye X., He J. (2017). Hypertension and breast cancer risk: A systematic review and metaanalysis. Sci. Rep..

[19] Chen S., Tao M., Zhang L.Z. (2017). The association between diabetes/hyperglycemia and the prognosis of cervical cancer patients: A systematic review and meta-analysis. Medicine.

[20] Pastakia S.D., Ali S.M., Kamano J.H., Akwanalo C.O., Ndege S.K., Buckwalter V.L., Vedanthan R., Bloomfield G.S. (2013). Screening for diabetes and hypertension in a rural low income setting in western Kenya utilizing home based and community-based strategies. Glob. Health.

[21] Mohan S., Jarhyan P., Ghosh S., Venkateshmurthy N.S., Gupta R., Rana R., Malhotra C., Rao M.B., Kalra S., Tandon N. (2018). UDAY: A comprehensive diabetes and hypertension prevention and management program in India. BMJ Open.

[22] Agency FDRoECS (2013). Population Projection of Ethiopia for All Regions At Wereda Level from 2014–2017.

[23] Federal Democratic Republic of Ethiopia Ministry of Health (2016). National Strategic Action Plan (NSAP) for Prevention & Control of Non Communicable Diseases in Ethiopia 2014–2016.

[24] Federal Democratic Republic of Ethiopia Ministry of Health (2015). Guideline for Cervical Cancer Prevention and Control in Ethiopia.

[25] Abeje S., Seme A., Tibelt A. (2019). Factors associated with breast cancer screening awareness and practices of women in Addis Ababa, Ethiopia. BMC Women Health.

[26] Getahun T., Kaba M., Derseh B.T. (2020). Intention to Screen for Cervical Cancer in Debre Berhan Town, Amhara Regional State, Ethiopia: Application of Theory of Planned Behavior. J. Cancer Epidemiol..

[27] Nwabichie C.C., Manaf R.A., Ismail S.B. (2018). Factors Affecting Uptake of Cervical Cancer Screening among African Women in Klang Valley, Malaysia. Asian Pac. J. Cancer Prev..

[28] Rodríguez-Gómez M., Ruiz-Pérez I., Martín-Calderón S., Pastor-Moreno G., Artazcoz L., Escribà-Agüir V. (2020). Effectiveness of patient-targeted interventions to increase cancer screening participation in rural areas: A systematic review. Int. J. Nurs. Stud..

[29] Tin K.N., Ngamjarus C., Rattanakanokchai S., Sothornwit J., Aue-aungkul A., Paing A.K., Pattanittum P., Jampathong N., Lumbiganon P. (2013). Interventions to increase the uptake of cervical cancer screening in lowand middle-income countries: A systematic review and meta-analysis. BMC Women Health.

[30] Popalis M.L., Ramirez S.I., Leach K.M., Granzow M.E., Stoltzfus K.C., Moss J.L. (2022). Improving cervical cancer screening rates: A scoping review of resources and interventions. Cancer Causes Control.

[31] Jones C.L., Jensen J.D., Scherr C.L., Brown N.R., Christy K., Weaver J. (2015). The Health Belief Model as an Explanatory Framework in Communication Research: Exploring Parallel, Serial, and Moderated Mediation. Health Commun..

[32] Ajzen I. (1991). The theory of planned behavior. Organ. Behav. Hum. Decis. Process..

[33] Ngigi S., Busolo D. (2018). Behaviour Change Communication in Health Promotion: Appropriate Practices and Promising Approaches. Int. J. Innov. Res. Dev..

[34] Abu S.H., Woldehanna B.T., Nida1 E.T., Tilahun A.W., Gebremariam M.Y., Sisay M.M. (2020). The role of health education on cervical cancer screening uptake at selected health centers in Addis Ababa. PLoS ONE.

[35] WHO (2019). Ethiopia Sets to Improve Hypertension Prevention and Control at Primary Health Care Level.

[36] Dozie U.W., Ebirim C.I., Dike C.R., Dozie I.N., Ibe S.N., Abanobi O.C. (2021). Determinants of cervical cancer screening uptake among female undergraduates in a tertiary institution in south eastern Nigeria: A cross sectional study. J. Prev. Med. Hyg..

[37] Gizaw M., Teka B., Ruddies F., Abebe T., Kaufmann A.M., Worku A., Wienke A., Jemal A., Addissie A., Kantelhardt E.J. (2019). Uptake of Cervical Cancer Screening in Ethiopia by Self-Sampling HPV DNA Compared to Visual Inspection with Acetic Acid: A Cluster Randomized Trial. Cancer Prev. Res..

[38] Mekonnen B.D. (2020). Cervical Cancer Screening Uptake and Associated Factors among HIV-Positive Women in Ethiopia: A Systematic Review and Meta-Analysis. Adv. Prev. Med..

[39] Lera T., Beyene A., Bekele B., Abreha S. (2020). Breast self-examination and associated factors among women in Wolaita Sodo, Ethiopia: A community-based crosssectional study. BMC Women Health.

[40] Ayenew A.A., Zewdu B.F., Nigussie A.A. (2020). Uptake of cervical cancer screening service and associated factors among age-eligible women in Ethiopia: Systematic review and meta-analysis. Infect. Agent Cancer.

[41] Assefa A.A., Abera G., Geta M. (2021). Breast Cancer Screening Practice and Associated Factors among Women Aged 20–70 Years in Urban Settings of SNNPR, Ethiopia. Breast Cancer Targets Ther..

[42] Yeshitila Y.G., Kassa G.M., Gebeyehu S., Memiah P., Desta M. (2021). Breast self-examination practice and its determinants among women in Ethiopia: A systematic review and meta-analysis. PLoS ONE.

[43] Tewelde B., Tamire M., Kaba M. (2022). Breast self-examination practice and predictors among female secondary school teachers in Addis Ababa, Ethiopia: Using the health belief model. BMC Women Health.

[44] Abamecha F., Tena A., Kiros G. (2019). Psychographic predictors of intention to use cervical cancer screening services among women attending maternal and child health services in Southern Ethiopia: The theory of planned behavior (TPB) perspective. BMC Public Health.

[45] Solomon K., Tamire M., Kaba M. (2019). Predictors of cervical cancer screening practice among HIV positive women attending adult anti-retroviral treatment clinics in Bishoftu town, Ethiopia: The application of a health belief model. BMC Cancer.

[46] WHO (2020). Integrated management of NCDs. WHO Package of Essential Noncommunicable (PEN) Disease Interventions for Primary Health Care.

