# Supplementary material for: Service-Delivery Models to Increase the Uptake of Non-Communicable Disease Screening in South-Central Ethiopia: A Difference-In-Differences Analysis
Source: Diseases. 2024 Nov 5;12(11):278. doi: 10.3390/diseases12110278 (PMC11593248; doi:10.3390/diseases12110278)
Supplement: Supplementary file 1 [file diseases-12-00278-s001.zip › diseases-3203854-supplementary.pdf]

Table S1. Socio-demographic characteristics, Knowledge, and Practice on NCD screening

1. Clinical breast examination			
**Section I Socio-demographic Characteristics**			
**Sr. No.**	**Questions**	**Response & Coding Categories**	**Skip**
101	How old are you?(In complete years)	________________	
102	What is your current marital status	1.Married/living together<br>2.Divorced/separated<br>3.Widowed<br>4.Never married/never lived together	
103	What is your current educational Status	1. Could not read and write<br>2. Read and write but no formal education<br>3. If formally educated, what is the last grade completed____________	
104	What is your current occupational status?	1. Government employee<br>2. Non-governmental organization employee<br>3. Housewife<br>4. Merchant<br>5. Farmer<br>6. Daily laborer<br>7. Student<br>8.Unemployed<br>9. Other, specify ________	
**Section II Knowledge on breast cancer**			
**Sr. No.**	**Questions**	**Response & Coding Categories**	**Skip**
105	Have you ever heard of breast cancer?	1. Yes →<br>2. No	Go to Q. 106
106	Breast cancer is transmittable disease	1. Yes<br>2. No<br>3. I don't know	
107	What are the risk factors for breast cancer? (multiple response is possible)	1. Positive family history of cancer<br>2. First child at late age (above 30 years old)<br>3. Early onset of menarche (under 12 years old)<br>4. Late menopause (above 55 years old)<br>5. Not breast feeding<br>6. Obesity<br>7. Alcohol consumption<br>8. Exposure to radiation<br>9. Having large breast<br>10. Punishment from God<br>11. I don't know<br>12. If other specify	

108	What are the sign and symptoms of breast cancer? (multiple response is possible)	1. Lump in the breast<br>2. Nipple Discharge<br>3. Changes in shape of the breast<br>4. Change in size of the breast<br>5. Swelling under the armpit<br>6. Pain in the breast<br>7. Dimpling of the breast<br>8. Discoloration of the breast<br>9. Inversion/Pulling in of nipple<br>10. I don't know<br>11. Other specify ……………	
**Section III clinical breast examination practice**			
**Sr. no.**	**Questions**	**Response & Coding Categories**	**Skip**
109	Have you ever had clinical breast examination?	1. Yes<br>2. No	
110	Have you take the clinical breast examination service today, at this facility?	1. Yes<br>2. No	
**2.Cervical cancer screening**			
**Section I Socio-demographic Characteristics**			
**Sr. No.**	**Questions**	**Response & Coding Categories**	**Skip**
201	How old are you?(In complete years)	________________	
202	What is your current marital status	1. Married/living together<br>2. Divorced/separated<br>3. Widowed<br>4. Never married/never lived together	
203	What is your current educational Status	1. Could not read and write<br>2. Read and write but no formal education<br>If formally educated, what is the last grade completed____________	
204	What is your current occupational status?	1. Government employee<br>2. Non-governmental organization employee<br>3. Housewife<br>4. Merchant<br>5. Farmer<br>6. Daily laborer<br>7. Student<br>8.Unemployed<br>9. Other, specify ________	
**Section II Knowledge on cervical cancer**			
**Sr. No.**	**Questions**	**Response & Coding Categories**	**Skip**
205	Have you ever heard of cervical cancer/cancer of the mouth/neck of the womb?	1. Yes →<br>2. No	206
206	What are the risk factors to cervical cancer? (multiple response is possible)	1. Early onset of sexual intercourse<br>2. Having multiple sexual partners<br>3. Family history of cervical cancer	

		4. Cigarette smoking<br>5. Having uncircumcised sexual partner<br>6. Don't know<br>Other, specify ________________	
207	What are the signs and symptoms of cervical cancer? (multiple response is possible)	1. Vaginal bleeding<br>2. Foul vaginal discharge<br>3. Pelvic or back pain<br>4. Post coital bleeding<br>5. Don't know<br>Other, specify_____________	
208	Do you think cervical cancer is preventable disease?	1. Yes →<br>2. No<br>3. Don't know	
209	How can we prevent cervical cancer? (multiple response is possible)	1. Avoid multiple sexual partners<br>2. Avoid early onset sexual intercourse<br>3. Quit smoking<br>4. Through vaccination<br>5. Undertake cervical cancer screening<br>6. Other, specify______________	
**Section III Cervical cancer screening practice**			
**Sr. no.**	**Questions**	**Response & Coding Categories**	**Skip**
210	Have you ever had cervical cancer screening?	1. Yes<br>2. No	
211	Have you take cervical cancer screening service today, at this facility?	1. Yes<br>2. No	
**3. Blood pressure measurement**			
**Section I Socio-demographic Characteristics**			
**Sr. No.**	**Questions**	**Response & Coding Categories**	**Skip**
301	How old are you?(In complete years)	_________________	
302	Sex	1. Male<br>2. Female	
303	What is your current marital status	1. Married/living together<br>2. Divorced/separated<br>3. Widowed<br>4. Never married/never lived together	
304	What is your current educational Status	1. Could not read and write<br>2. Read and write but no formal education<br>3. If formally educated, what is the last grade completed_____________	
305	What is your current occupational status?	1. Government employee<br>2. Non-governmental organization employee<br>3. Housewife<br>4. Merchant<br>5. Farmer<br>6. Daily laborer<br>7. Student<br>8.Unemployed	

		9. Other, specify ________	
**Section II. Knowledge on high blood pressure**			
**SN**	**Questions**	**Response & Coding Categories**	**Skip**
306	Have you ever heard of hypertension/ high blood pressure?	1. Yes →<br>2. No	Go to Q. 307
307	High blood pressure can transmit from one person to another?	1. Yes<br>2. No	
308	What is/are the risk factor/s for developing high blood pressure? (multiple responses are possible)	1. Hereditary<br>2. Smoking<br>3. Obesity<br>4. High fat intake<br>5. Excess alcohol intake<br>6. High salt intake<br>7. Don't know<br>Other (specify)…………	
309	What are the signs and symptoms of high blood pressure? (multiple responses are possible)	1. Asymptomatic<br>2. Headache<br>3. Palpitations<br>4. Poor vision<br>5. Dizziness<br>6. Don't know<br>7. Other (specify)………	
310	What is the normal range of blood pressure?	1. Less than 120/80<br>2. Less than 130/90<br>3. Less than 160/100<br>4. More than or equal to 160/100<br>5. Don't know	
311	How can you prevent/ control high blood pressure? (multiple responses are possible)	1. Minimize salt intake<br>2. Reduce fatty foods<br>3. Avoid excess alcohol<br>4. Avoid smoking<br>5. Regular exercise<br>6. Taking antihypertensive<br>7. Taking blood pressure measurement<br>8. Don't know<br>9. Other (Specify)…………..	
**Section III Blood pressure measurement practice**			
**Sr. no.**	**Questions**	**Response & Coding Categories**	**Skip**
312	Have you ever had your blood pressure measured by a doctor or other health worker?	1. Yes<br>2. No	

313	Have taken the blood pressure measurement service today, at this health facility?	1. Yes<br>2. No	
**4.Blood glucose measurement**			
**Section I Socio-demographic Characteristics**			
**Sr. No.**	**Questions**	**Response & Coding Categories**	**Skip**
401	How old are you?(In complete years)	________________	
402	Sex	1. Male<br>2. Female	
403	What is your current marital status	1. Married/living together<br>2. Divorced/separated<br>3. Widowed<br>4. Never married/never lived together	
404	What is your current educational Status	1. Could not read and write<br>2. Read and write but no formal education<br>3. If formally educated, what is the last grade completed____________	
405	What is your current occupational status?	1. Government employee<br>2. Non-governmental organization employee<br>3. Housewife<br>4. Merchant<br>5. Farmer<br>6. Daily laborer<br>7. Student<br>8.Unemployed<br>9. Other, specify _________	
**Section II. Knowledge on diabetes mellitus**			
**Sr. No.**	**Questions**	**Response & Coding Categories**	**Skip**
406	Have you ever heard of diabetic mellitus?	1. Yes →<br>2. No	407
407	What is/are the risk factor/s for developing diabetic mellitus? (multiple responses are possible)	1. Family history of diabetic mellitus<br>2. Being overweight/ obesity<br>3. Eating too much sugar<br>4. Sedentary life (or not getting enough exercise)<br>5. Stress<br>6. Don't know<br>7. Other (specify)…………	
408	What are the signs and symptoms of diabetic mellitus? (multiple responses are possible)	1. Increased thirst<br>2. Poor appetite<br>3. Frequent urination<br>4. Abdominal pain<br>5. Palpitation (due to high blood sugar)<br>6. Slow healing of cuts and wounds<br>7. Don't know<br>8. Other (specify)………	

408	Which of the following therapies are effective in controlling diabetes mellitus? (multiple responses are possible)	1. Insulin injection<br>2. Oral medications<br>3. Regular Exercise<br>4. Avoiding sugary foods<br>5. Regular eating of (herbs, ginger and cinnamon)<br>6. Taking blood glucose check up<br>7. Don't know<br>8. Other (specify)………………..	
**Section III blood glucose measurement practice**			
**Sr. No.**	**Questions**	**Response & Coding Categories**	**Skip**
409	Have you ever had blood sugar measurement by a doctor or other health worker?	1. Yes<br>2. No	
410	Have you take blood glucose measurement service today, at this facility?	1. Yes<br>2. No	
